# Lithium chloride promotes host resistance against *Pseudomonas aeruginosa* keratitis

**Published:** 2013-07-19

**Authors:** Kang Chen, Yongjian Wu, Min Zhu, Qiuchan Deng, Xinxin Nie, Meiyu Li, Minhao Wu, Xi Huang

**Affiliations:** 1 Department of Immunology, Institute of Human Virology, Zhongshan School of Medicine, Sun Yat-sen University, Guangzhou, China; 2 State Key Laboratory of Ophthalmology, Zhongshan Ophthalmic Center, Sun Yat-sen University, Guangzhou, China; 3 Key Laboratory of Tropical Diseases Control (Sun Yat-sen University), Ministry of Education, Guangzhou, China

## Abstract

**Purpose:**

To explore the role of lithium chloride (LiCl) in *Pseudomonas aeruginosa* (PA) keratitis.

**Methods:**

B6 mice were subconjunctivally injected with LiCl in contrast to appropriate control sodium chloride (NaCl), and then routinely infected with PA. Clinical score, slit-lamp photography, hematoxylin and eosin (H&E) staining, and bacterial plate counts were used to determine the role of LiCl in PA keratitis. Messenger ribonucleic acid and protein levels of inflammatory cytokines in PA-challenged mouse corneas and in vitro cultured macrophages and neutrophils were measured with real-time PCR and enzyme-linked immunosorbent assay (ELISA), respectively. Apoptosis of the infiltrating inflammatory cells in the PA-infected murine corneas was assessed using terminal deoxynucleotidyl transferase-mediated uridine 5′-triphosphate-biotin nick end labeling staining and propidium iodide staining associated with flow cytometry. In cultured murine macrophages and neutrophils, cell apoptosis was determined with annexin V/propidium iodide double staining associated with flow cytometry and western blot analysis for cleaved caspase-3 and cleaved poly(ADP-ribose) polymerase.

**Results:**

Treatment with LiCl reduced the severity of corneal disease by reducing corneal inflammatory response and bacterial burden. Moreover, LiCl increased anti-inflammatory cytokine interleukin-10 levels, decreased proinflammatory cytokine tumor necrosis factor-α levels, and enhanced apoptosis of infiltrating macrophages and neutrophils in the PA-infected mouse corneas. In vitro studies further confirmed that LiCl elevated anti-inflammatory cytokine expression but reduced proinflammatory cytokine production, as well as promoted cell apoptosis in murine macrophages and neutrophils.

**Conclusions:**

This study demonstrates a protective role of LiCl in PA keratitis. LiCl promotes host resistance against PA infection by suppressing inflammatory responses, enhancing inflammatory cell apoptosis, and promoting bacterial clearance.

## Introduction

Microbial keratitis induced by *Pseudomonas aeruginosa* (PA) is a rapidly progressive ocular disease characterized by a suppurative stromal infiltrate with a marked mucopurulent exudate, and often causes corneal perforation within several days postinfection [1,2]. Innate immunity is the first line of host defense against microbial infection. Upon recognition of invading microbes by pattern-recognition receptors, inflammatory cells, including polymorphonuclear neutrophils and macrophages, are recruited to the infection sites [3,4]. These inflammatory phagocytes not only directly kill the invading bacteria but also produce various proinflammatory cytokines (e.g., interleukin [IL]-6, IL-1β, tumor necrosis factor [TNF]-α) and anti-inflammatory cytokines such as IL-10 to modulate the antibacterial immunity [5]. However, if uncontrolled, these inflammatory mediators often elicit an overly robust response, favoring bystander tissue damage [1,6,7]. Thus, tight regulation of host inflammatory responses, especially the number and activity of infiltrating inflammatory phagocytes, is critical for disease resolution of PA keratitis [8].

Programmed cell death (apoptosis) is an important strategy for controlling host inflammatory responses, but the role of apoptosis in PA keratitis remains controversial. Dr. Hazlett’s group found that Fas ligand (FasL) knockout mice displayed more severe disease than wild-type mice after PA corneal infection [9], and demonstrated that treatment with substance P, an antiapoptotic neuropeptide, accelerates the disease progression of PA keratitis [10]. However, Dr. Pearlman’s laboratory reported that bacterial virulence factors such as exoenzyme S and exoenzyme T promote neutrophil apoptosis and bacterial survival, leading to the aggravation of the severity of PA keratitis [11].

As one of the lightest chemical compounds, lithium chloride (LiCl) has been used as a mood stabilizer for treating diseases such as bipolar disorder for many years [12]. Studies have demonstrated that LiCl is capable of modulating various biologic processes, such as glycogen synthesis, gene expression, mobility, apoptosis, and inflammation [13]. For example, LiCl inhibits the activation of glycogen synthase kinase 3β (GSK3β) [14], and enhances the activation of wingless (Wnt)/β-catenin signaling [14]. LiCl also influences the second messenger system by modulating the activity of the enzymes involved in the metabolic processes of inositol 1,4,5-triphosphate (IP3) [15], diacylglycerol (DAG) [16], and cyclic adenosine monophosphatase level (c-AMP) [17]. Furthermore, it has been reported that LiCl functions as an inflammatory suppressor in dermatitis [18], lipopolysaccharide (LPS)-induced inflammation [19], toll-like receptor (TLR)-mediated chronic intestinal inflammation [20], and *Francisella* infection [21]. LiCl also functions to modulate apoptosis of various cell types, including macrophages [22], neuron cells [23], lymphoid cells [24], and epithelial cells [25-27]. To date, LiCl exhibits great potential in treating Alzheimer disease [28], bipolar disorder [29], diabetes [30], and serous ovarian cancer [31]. However, the role of LiCl in ocular infection remains to be determined.

In this study, we demonstrated that treatment with LiCl in B6 mice promoted host resistance against PA corneal infection, by reducing host inflammatory responses and bacterial burden. Furthermore, in vivo and in vitro studies suggested that LiCl enhanced the production of anti-inflammatory cytokines, inhibited the expression of proinflammatory cytokines, and promoted apoptosis of inflammatory cells. These data together indicate that LiCl may be a potential therapeutic strategy for PA keratitis, as well as other microbial keratitis.

## Methods

### Ocular infection and clinical examination

Eight-week-old female C57BL/6 (B6) mice were purchased from the Animal Supply Center of Sun Yat-sen University Zhongshan School of Medicine. Mice were anesthetized with inhalational anaesthetic, diethyl ether (with a concentration of 1.9%) and placed beneath a stereoscopic microscope at 40X magnification. The cornea of the left eye was wounded with three 1 mm incisions using a sterile 25 gauge needle. A 5 µl aliquot containing 1×10^6^ colony-forming units of PA stain American Type Culture Collection (ATCC; Manassas, VA) 19660 was topically applied to the ocular surface. Eyes were examined at 1 day postinfection (p.i.) and/or at times described below, to ensure that mice were similarly infected and to monitor the disease. Corneal disease was graded using an established scale [32]: 0, clear or slight opacity partially or fully covering the pupil; +1, slight opacity partially or fully covering the anterior segment; +2, dense opacity partially or fully covering the pupil; +3, dense opacity covering the entire anterior segment; and +4, corneal perforation or phthisis. All animal experiments were performed in accordance with the National Institutes of Health Guide for the Care and Use of Laboratory Animals.

### Cell culture

Murine macrophage-like RAW264.7 cells (ATCC #TIB-71) were cultured in Dulbecco's Modified Eagle's Medium (DMEM; Invitrogen, Carlsbad, CA) media supplemented with 10% (v/v) fetal bovine serum (Invitrogen, Carlsbad, CA), 1% penicillin-streptomycin (Invitrogen), and 1% L-glutamine (Invitrogen) at the permissive temperature of 37 °C.

### Isolation and culture of mouse bone marrow–derived neutrophils

Bone marrow–derived neutrophils (briefly called neutrophils) were isolated from 6-week-old female B6 mice as described by others [33]. Briefly, marrow cells were flushed from the femur and tibia with ice-cold Roswell Park Memorial Institute (RPMI)-1640 media (Invitrogen) media, and then rinsed and treated with erythrocyte lysis buffer to remove the red blood cells. Resuspended cells were laid on the top of discontinuous Percoll density gradients (52%, 64%, 72%) and centrifuged at 400 ×g for 30 min at room temperature. Neutrophils were isolated from the bottom layer (64%–72%), counted, and plated on a 12-well culture plate. Cells were cultured in Rosewell Park Memorial Institute 1640 media supplemented with 10% (v/v) fetal bovine serum, 1% penicillin-streptomycin, and 1% L-glutamine. Trypan blue (Invitrogen) exclusion indicated that the cell viability was approximately 95%. Microscopic evaluation demonstrated that more than 95% of the isolated cells were neutrophils.

### Lithium chloride interference in vivo and in vitro

LiCl (Sigma, St. Louis, MO) or appropriate control NaCl (Sigma) was subconjunctivally injected into the left eye of B6 mice (5 µl/mouse at a concentration of 0.15 M, n=5/group/time) 1 day before infection and then topically applied to the infected corneas (5 µl/mouse per time at an isotonic concentration of 0.15 M, once on the day of infection, and twice on both 1 and 3 days p.i.) [34]. For in vitro interference, murine macrophage-like RAW264.7 cells and mouse bone marrow–derived neutrophils were cultured in the media with additional 10 mM LiCl versus NaCl [35].

### Real-time polymerase chain reaction

Total RNA was isolated from individual corneas or cell pellets by using TRIzol (Invitrogen). One µg of total RNA was reversely transcribed to produce cDNA, and then amplified using SYBR Green Master Mix (Bio-Rad, Hercules, CA) following the manufacturer’s protocol. Primer sequences for IL-6, IL-1β, TNF-α, IL-10, and β-actin are listed in Table 1. Quantitative real-time PCR reactions were performed using the CFX96 Real-Time PCR System (Bio-Rad). Relative messenger ribonucleic acid (mRNA) levels were calculated after normalization to β-actin.

**Table 1:** Nucleotide sequence of the speciﬁc primers used in PCR ampliﬁcation.

**Gene**	**Primer sequence (5′-3′)**
β-actin	F: GATTACTGCTCTGGCTCCTAGC
	R: GACTCATCGTACTCCTGCTTGC
IL-6	F: CACAAGTCCGGAGAGGAGAC
	R: CAGAATTGCCATTGCACAAC
IL-1β	F: CGCAGCAGCACATCAACAAGAGC
	R: TGTCCTCATCCTGGAAGGTCCACG
TNF-α	F: CACAGAAAGCATGATCCGCGAC
	R: TGCCACAAGCAGGAATGAGAAGAG
IL-10	F: AGCTGGACAACATACTGCTAACCGAC
	R: CTTGATTTCTGGGCCATGCTTCTCTG

### Western blot analysis

For corneal expression of P-GSK3β (Ser9) and β-catenin, whole corneas (n=5/group/time) were collected and pooled from the LiCl- versus NaCl-treated B6 eyes before infection and at 1 day p.i. Pooled corneas were lysed and homogenized using a 1 ml glass tissue homogenizer in lysis buffer containing 1 mM phenylmethylsulfonyl ﬂuoride, 1% (v/v) protease inhibitor cocktail, and 1 mM Dithiothreitol (DTT; all purchased from Sigma). For in vitro detection, RAW264.7 cells or bone marrow derived neutrophils were washed three times with ice-cold phosphate buffered saline (PBS, pH=7.4, Invitrogen) and then treated with the lysis buffer. Protein concentration of the supernatant was determined with the Quick Start Bradford protein assay (Bio-Rad). Thirty µg of each sample was loaded, separated on 10% sodium dodecyl sulfate–polyacrylamide gel electrophoresis, and then transferred to a supported nitrocellulose membrane (Pall Life Sciences, Ann Arbor, MI). Blots were blocked in 5% non-fat dry milk in PBS with 0.1% Tween 20 (PBST) and incubated with the primary rabbit monoclonal antibodies (Abs) for P-GSK3β (Ser9), β-catenin, cleaved caspase-3, and cleaved poly(ADP-ribose) polymerase (PARP; 1:1000; Cell Signaling, Carlsbad, CA) at 4 °C overnight, followed by incubation with secondary IRDye 800CW Donkey antirabbit immunoglobulin (IgG) (H + L) Ab (1:5000, LI-COR Biosciences, Lincoln, NE) for 1 h. Finally, blots were detected using the Odyssey Infrared Imaging System (LI-COR Biosciences) according to the manufacturer’s protocol.

### Terminal deoxynucleotidyl transferase-mediated uridine 5′-triphosphate-biotin nick end labeling assay and hematoxylin and eosin staining

Uninfected and infected eyes from the LiCl- versus NaCl-treated mice (n=3/group/time) were enucleated at 5 days p.i. for terminal deoxynucleotidyl transferase transferase-mediated uridine 5′-triphosphate-biotin nick end labeling (TUNEL) staining with a terminal deoxynucleotidyl transferase (TdT) kit (Promega, Madison, WI) according to the manufacturer’s instruction. Eyes were ﬁxed in a 3.7% formaldehyde solution (Sigma) and embedded in parafﬁn. Eight-μm-thick sections were cut, deparafﬁnized, rehydrated, and rinsed with DNase-free water (Invitrogen). Sections were permeabilized using proteinase K solution (20 μg/ml, Sigma) for 15 min and then fixed again using 3.7% formaldehyde solution (Sigma). Each section was incubated with TdT incubation buffer, which contains 45 μl equilibration buffer, 5 μl nucleotide mix, and 1 μl TdT enzyme at 37 °C for 1 h to label the DNA nick ends, and then incubated with 4,6-diamino-2-phenyl indole (DAPI, 1:10,000, Sigma) for nuclear staining. Control samples were treated similarly but without TdT enzyme treatment. For histopathology in the B6 mouse eyes, sections were H&E stained as described by others [36]. Briefly, eyes were ﬁxed in a 3.7% formaldehyde solution (Sigma) and embedded in parafﬁn. Eight-μm–thick sections were cut, deparafﬁnized, rehydrated, and rinsed with distilled water. Then, sections were stained with Hematoxylin for 5 min, differentiated with 1% HCl in 70% alcohol for 30 s, and washed with tape water for 15 min. Finally, sections were stained with Eosin for 2 min, dehydrated, and mounted. All sections were visualized with a Carl Zeiss microscope (Jena, Germany).

### Flow cytometry

Flow cytometric analysis was performed as described by others [5,37]. For corneal cells, five corneas were pooled and digested in collagenase type I (Sigma). Cell suspensions were filtered, centrifuged, and resuspended in PBS with 2% bovine serum albumin (BSA). After blocking, cells were distributed into 100 μl samples and incubated with the following Abs for 30 min on ice: allophycocyanin (APC)-conjugated anti-Gr-1 (BD, Sparks, MD), Alexa Fluor 488-conjugated anti-F4/80 (Invitrogen), or isotype control APC-conjugated antirat IgG2b (BD) and Alexa Fluor 488-conjugated antirat IgG2a (Invitrogen). Then the cells were washed and resuspended in 500 μl binding buffer, followed by the addition of 5 μl propidium iodide (PI). Finally, cell suspensions were incubated at room temperature away from light for 15 min, and analyzed with flow cytometry (Beckman Coulter EPICS XL/MCL, Fullerton, CA). For in vitro cultured cells, cell apoptosis was assessed with flow cytometry using an annexin V-fluorescein isothiocyanate apoptosis detection kit (BD) according to the manufacturer’s instructions. Briefly, cells were pooled, washed, and resuspended in 500 μl binding buffer, followed by addition of 5 μl annexin V-fluorescein isothiocyanate and 5 μl PI. Then, cells were incubated at room temperature away from light for 15 min, and analyzed with flow cytometry (Beckman Coulter EPICS XL/MCL). Viable cells were unstained with annexin V or PI, early apoptotic cells were stained with annexin V but not PI, and late apoptotic cells were stained with annexin V and PI.

### Enzyme-linked immunosorbent assay

For the in vivo studies, corneas from LiCl- versus NaCl-treated B6 mice (n=5/group/time) were individually collected at 1 and 5 days p.i., and then homogenized in 0.5 ml of PBS with 0.1% Tween-20. For the in vitro studies, cell supernatant of LiCl- versus NaCl-treated RAW264.7 cells or neutrophils was collected at 6 h post challenge. The protein levels of IL-10 and TNF-α were tested using enzyme-linked immunosorbent assay (ELISA) kits (R&D; Minneapolis, MN) according to the manufacturer’s instructions. The reported sensitivity of these assays is <4.8 pg/ml for IL-10 and <5.1 pg/ml for TNF-α.

### Bacterial plate counts

Corneas from LiCl- versus NaCl-treated B6 mice were collected at 1 and 5 days p.i. (n=5/group/time). The number of viable bacteria was quantitated as previously described [38]. Individual corneas were homogenized in normal saline solution containing 0.25% BSA. Serial ten-fold dilutions of the samples were plated on *Pseudomonas* isolation agar (BD Difco Laboratories, Sparks, MD) in triplicate, and the plates were incubated overnight at 37 °C. Results are reported as log_10_ colony-forming units per cornea±standard error of the mean (SEM).

### Statistical analysis

The differences in clinical scores between the LiCl- versus the NaCl-treated B6 corneas were tested with the Mann–Whitney U test at 1, 3, and 5 days p.i. An unpaired, two-tailed Student *t* test was used to determine the significance of the other assays. Data were considered significant at p<0.05.

## Results

### Lithium chloride promoted host resistance to *Pseudomonas aeruginosa* keratitis

**Figure 1:**
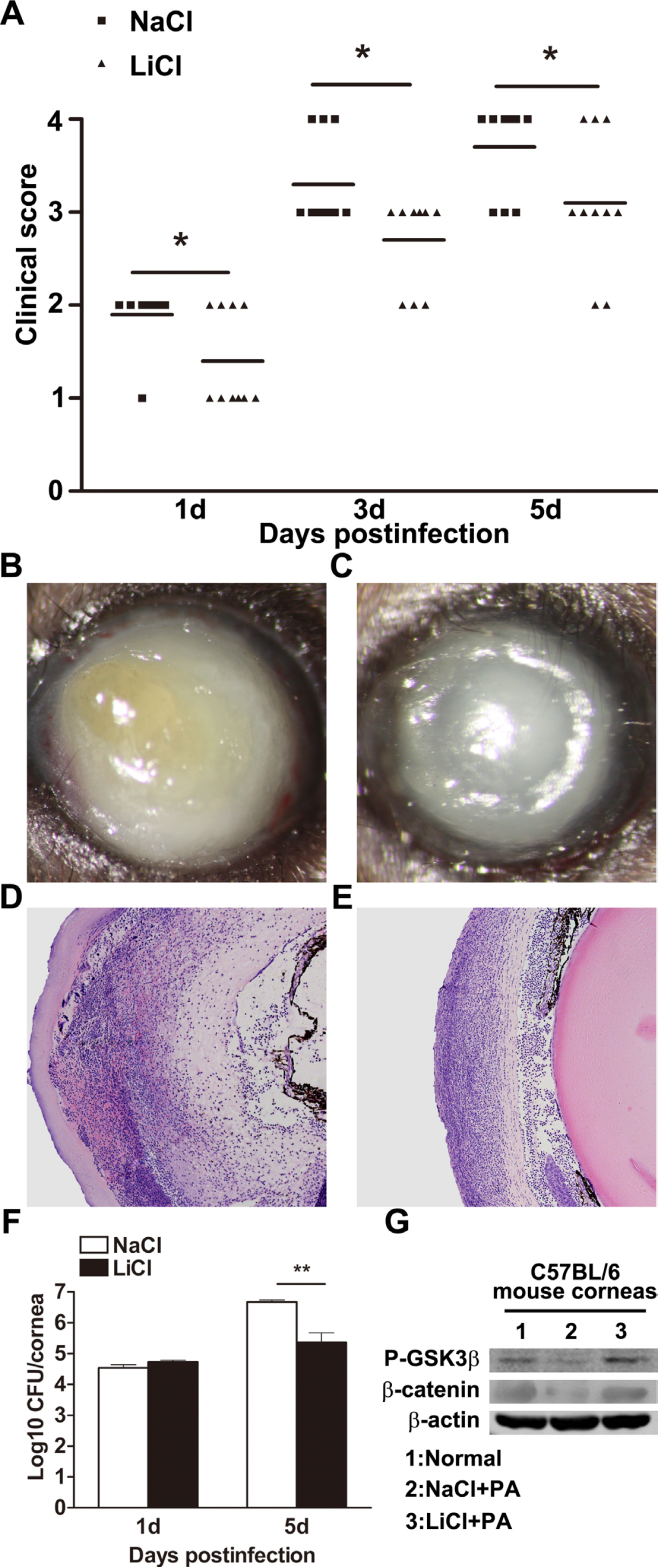
LiCl promotes host resistance against *Pseudomonas aeruginosa* (PA) keratitis. **A**: Clinical scores showed statistically significant differences in lithium chloride– (LiCl-) versus sodium chloride (NaCl)–treated corneas at 1, 3, and 5 days p.i. The horizontal lines among the open and filled dots represent the means of the indicated clinical scores. Three individual experiments were performed, each with ten animals/group/time. Data were generated from one representative experiment. Slit-lamp photographs of *Pseudomonas aeruginosa* (PA)–infected eyes at 5 days p.i. displayed reduced disease severity in LiCl-treated (**C**) versus NaCl-treated (**B**) mice. Hematoxylin and eosin (H&E) staining was used to examine the histopathology of infected eyes at 5 days p.i. after treatment with LiCl (**E**) versus NaCl (**D**). Magnification=100X. Images shown are representative of three individual experiments each with three mice per group. **F**: Plate count assay data show that bacterial burden was decreased in infected B6 corneas at 5 days p.i. after treatment with LiCl versus NaCl. Data are the mean±standard error of the mean (SEM) and represent three individual experiments each with five corneas/group/time/assay. **G**: The protein levels of P-glycogen synthase kinase 3β (P-GSK3β) and β-catenin in LiCl- versus NaCl-treated B6 corneas were detected with western blot to confirm the efficacy of LiCl treatment. Data shown represent one of three individual experiments, each using five pooled corneas/time.* p<0.05; **, p<0.01.

To determine the potential role of LiCl in PA keratitis, B6 mice were subconjunctivally injected with LiCl and NaCl (an appropriate control). Clinical score data showed that the LiCl-treated B6 mice exhibited less disease severity at 1, 3, and 5 days p.i. (Figure 1A, p<0.05, p<0.05, and p<0.05, respectively). Representative photographs of the LiCl-treated B6 corneas (Figure 1C) show less opacity than the NaCl-treated corneas (Figure 1B) at 5 days p.i. We also enucleated the infected eyes at 5 days p.i. from LiCl- versus NaCl-treated B6 mice for histopathology. H&E staining data indicated that LiCl-treated B6 corneas (Figure 1E) were much thinner and less swollen, with fewer infiltrated inflammatory cells in the stroma and anterior chamber, in contrast to the NaCl-treated corneas (Figure 1D). Bacterial plate counts were used to detect viable bacteria in the infected cornea of the LiCl- versus NaCl-treated mice at 1 and 5 days p.i. The results showed that LiCl treatment decreased the bacterial load at 5 days p.i. (Figure 1F, p<0.01), while no change was shown between the two groups at 1 day p.i. The efficacy of in vivo use of LiCl was confirmed with western blot (Figure 1G). The results showed that subconjunctival injection of LiCl dramatically upregulated the protein levels of P-GSK3β and β-catenin, indicating the efficacy of LiCl. These results together demonstrated that LiCl promoted host resistance to PA corneal infection.

### Lithium chloride modulated pro- and anti-inflammatory cytokine expression in vivo

**Figure 2:**
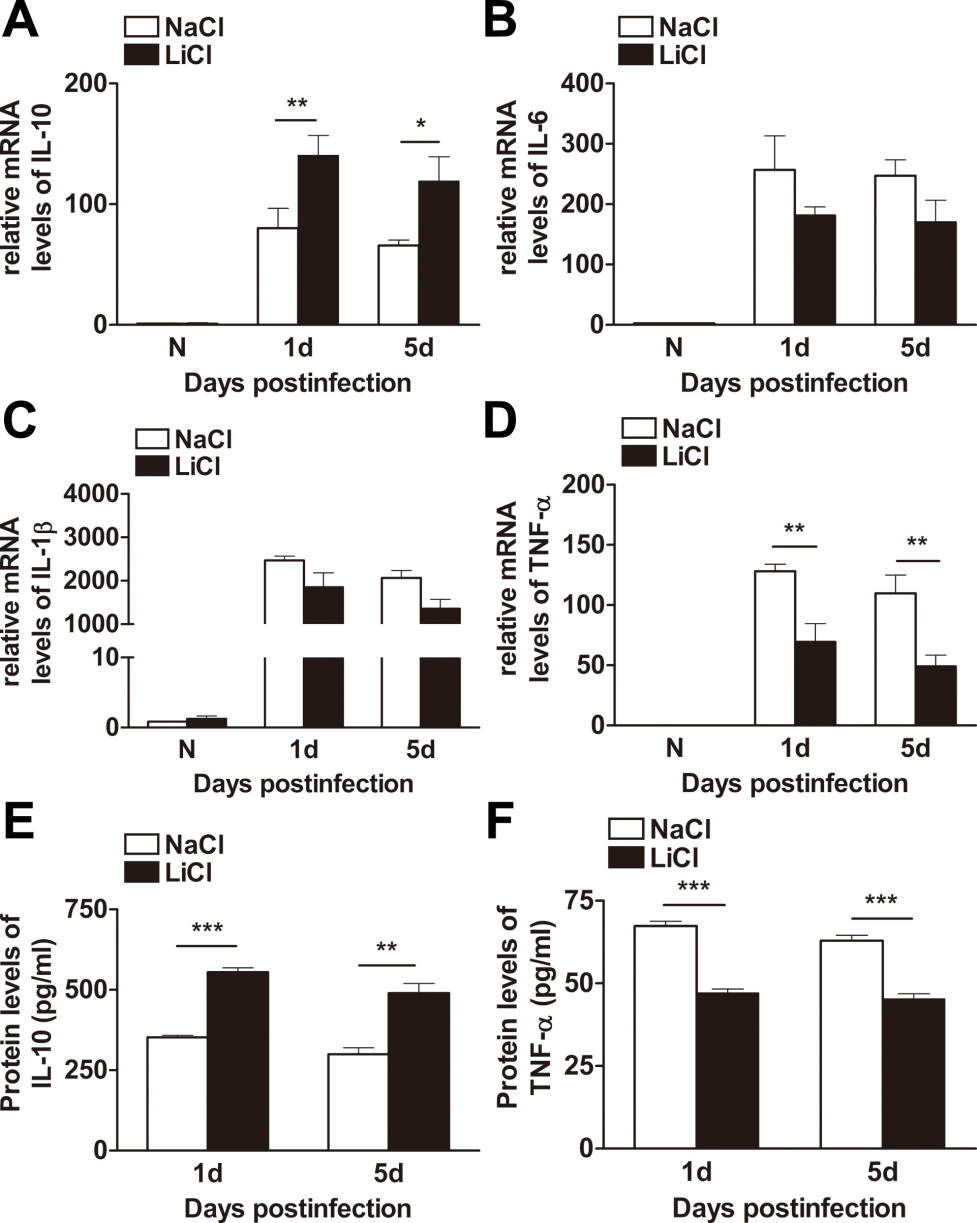
Lithium chloride regulates inflammatory cytokine production in vivo. Relative messenger ribonucleic acid (mRNA) expression levels (fold changes) of interleukin-10 (IL-10) (**A**), IL-6 (**B**), IL-1β (**C**), and tumor necrosis factor-alpha (TNF-α) (**D**) in lithium chloride– (LiCl-) versus sodium chloride (NaCl)–treated corneas at 1 and 5 days p.i. were tested using real-time polymerase chain reaction (PCR). Protein levels of IL-10 (**E**) and TNF-α (**F**) in LiCl- versus NaCl-treated corneas at 1 and 5 days p.i. were tested using enzyme-linked immunosorbent assay (ELISA). Data are the mean±standard error of the mean (SEM) and represent three individual experiments with five animals/group/time. *, p<0.05; **, p<0.01; ***, p<0.001.

To explore the mechanism by which LiCl promoted host resistance against PA infection, we examined the expression of selected pro- and anti-inflammatory cytokines with real-time PCR in LiCl- versus NaCl-treated corneas. At 1 and 5 days p.i., treatment with LiCl enhanced the mRNA levels of IL-10 (Figure 2A, p<0.01, p<0.05, at 1 and 5 days p.i., respectively) and suppressed TNF-α expression (Figure 2D, both p<0.01), while the mRNA levels of proinflammatory cytokines IL-6 and IL-1β were unchanged between the two groups (Figure 2B,C). Furthermore, LiCl significantly upregulated the protein levels of IL-10 (Figure 2E, p<0.001, p<0.01, at 1 and 5 days p.i., respectively) but downregulated the protein levels of TNF-α (Figure 2F, both p<0.001). These data suggested that LiCl inhibited inflammatory responses in PA keratitis via increasing the expression of anti-inflammatory cytokine IL-10 and decreasing the expression of proinflammatory cytokine TNF-α.

### Lithium chloride promoted cell apoptosis in vivo

**Figure 3:**
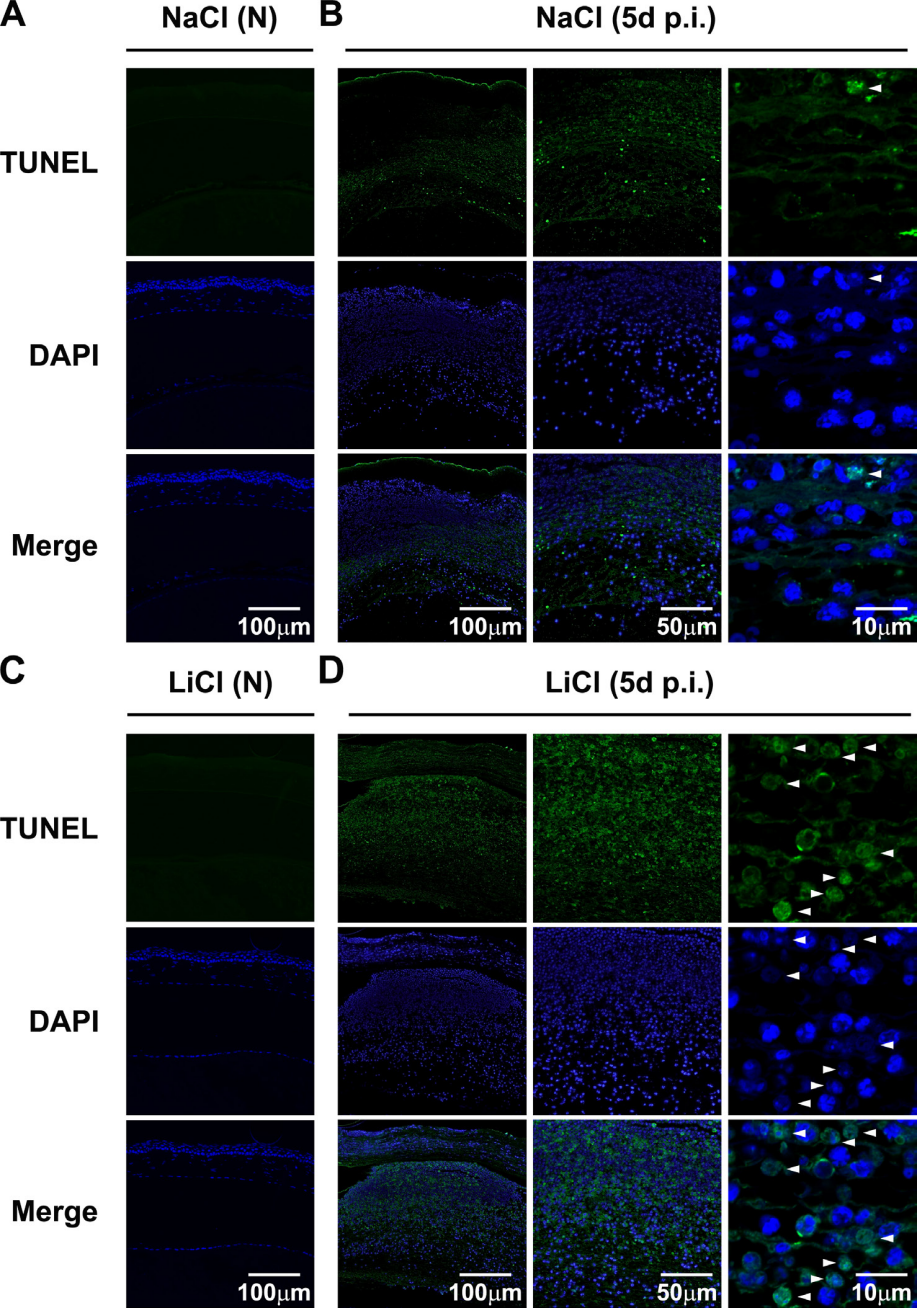
Apoptosis in the infected cornea assessed with terminal deoxynucleotidyl transferase-mediated uridine 5′-triphosphate-biotin nick end labeling staining. Terminal deoxynucleotidyl transferase-mediated uridine 5′-triphosphate-biotin nick end labeling (TUNEL)-positive staining (green) was detected in lithium chloride (LiCl)– (**C** and **D**) versus sodium chloride (NaCl)–treated(**A** and **B**) corneas before (N) and at 5 days p.i. Cell nuclei were stained with 4,6-diamino-2-phenyl indole (DAPI; blue). Apoptotic cells were determined by colocalization of TUNEL and DAPI-positive staining (as indicated by white arrows). Magnification=100X, 200X, and 630X, respectively. Images shown are representative of three individual experiments each with three mice per group.

Apoptosis in LiCl- versus NaCl-treated B6 corneas was assessed with TUNEL staining before and at 5 days p.i. (Figure 3A–D).

No TUNEL-positive cells were detected in the NaCl-treated (Figure 3A) and LiCl-treated corneas before infection (Figure 3C). However, at 5 days p.i., in contrast to the NaCl-treated infected corneas (Figure 3B), the LiCl-treated infected corneas (Figure 3D) showed more intense TUNEL-positive staining in the cornea stroma (magnification=100X, 200X). Confocal data (magnification=630X) further demonstrated that colocalization (indicated with white arrows) of nuclear DAPI staining (blue) and TUNEL staining (green) was dramatically increased in the LiCl-treated corneas (Figure 3D) compared with the NaCl-treated corneas (Figure 3B), suggesting that LiCl promoted apoptosis of the infiltrating cells in PA-infected corneas.

### Lithium chloride promoted inflammatory cell apoptosis in *Pseudomonas aeruginosa–*infected corneas

**Figure 4:**
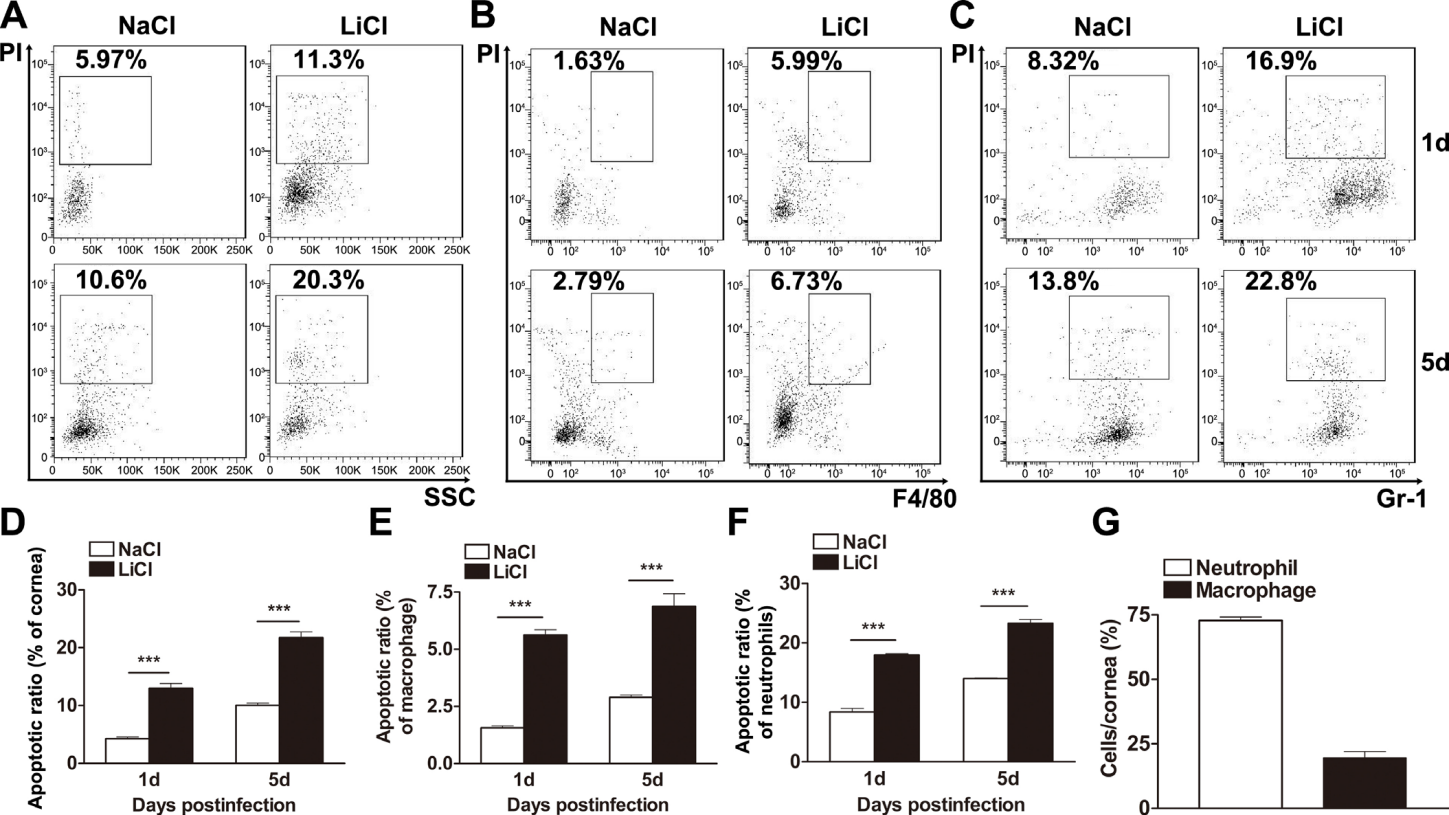
Apoptosis in the infected corneas assessed with flow cytometry. **A**: The apoptotic ratios in lithium chloride– (LiCl-) versus sodium chloride (NaCl)–treated corneas at 1 and 5 days p.i. were detected with propidium iodide (PI) staining associated with flow cytometry, as calculated by the average percentage of PI-positive cells in the infected cornea (**D**). The respective apoptotic ratios of macrophages (**B**) and neutrophils (**C**) in LiCl- versus NaCl-treated corneas at 1 and 5 days p.i. were analyzed with flow cytometry associated with triple staining for PI, F4/80, and Gr-1, as calculated by the average percentage of PI-positive cells in either F4/80-positive macrophages (**E**) or Gr-1-positive neutrophils (**F**). **G**: The percentages of the macrophages and neutrophils in the corneal infiltrating cells were determined with F4/80 and Gr-1 staining associated with flow cytometry. Data are the mean±standard error of the mean (SEM) and represent three individual experiments (n=5). ***, p<0.001.

To determine the apoptotic ratio of corneal cells in the LiCl- versus NaCl-treated PA-infected mice, corneal cells were analyzed with PI staining associated with flow cytometry. The results showed that in the NaCl-treated B6 corneas, PI-positive cells accounted for 5.97% and 10.6% at 1 and 5 days p.i., respectively (Figure 4A), while in the LiCl-treated B6 corneas, the percentage of apoptotic cells was elevated to 11.3% and 20.3% at 1 and 5 days p.i., respectively (Figure 4A), as calculated by the average percentage of PI-positive cells in B6 cornea cell suspensions (Figure 4D, both p<0.001 at 1 and 5 days p.i.).

Furthermore, corneal cells were analyzed by using F4/80 and Gr-1 double staining associated with flow cytometry, to identify the cell types of the infiltrating cells. The results showed that in the infected B6 corneas, the percentage of neutrophils (Gr-1 positive) and macrophages (F4/80 positive) was 75% and 20%, respectively (Figure 4G), while other cells (as shown in Gr-1 negative and F4/80 negative), including dendritic cells, resident corneal fibroblasts, corneal epithelial cells, and so on, accounted for only 5% of corneal cells (data not shown). In addition, cell apoptosis in macrophages and neutrophils was analyzed with flow cytometry in each cell population, and the results showed that treatment with LiCl significantly increased the apoptotic ratio of macrophages (Figure 4B) and neutrophils (Figure 4C) at 1 and 5 days p.i., as calculated by the average percentage of PI-positive cells in F4/80 positive cells (Figure 4E, both p<0.001 at 1 and 5 days p.i.) or Gr-1 positive cells (Figure 4F, both p<0.001 at 1 and 5 days p.i.).

### Lithium chloride modulated pro- and anti-inflammatory cytokine expression in vitro

**Figure 5:**
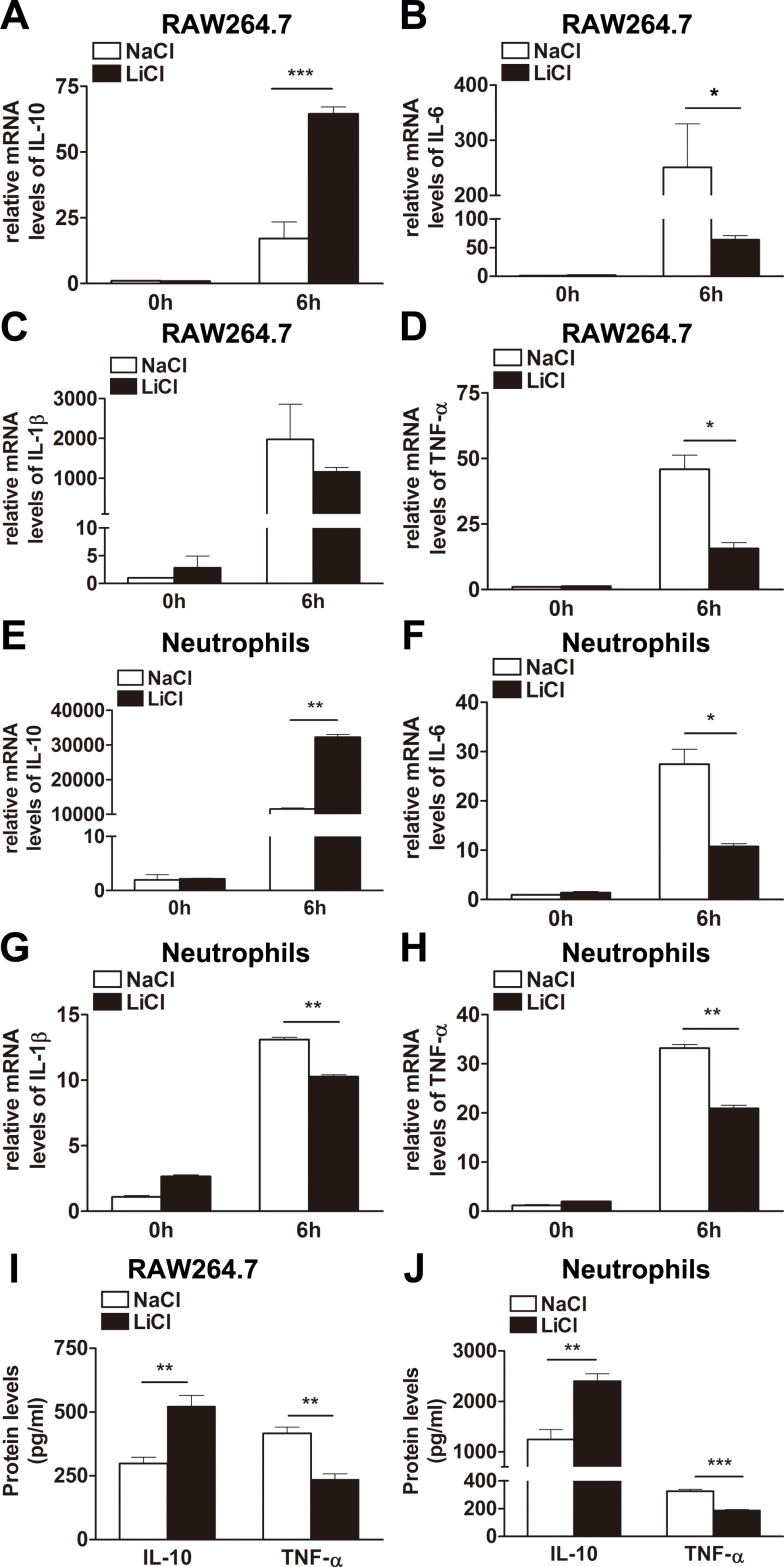
Lithium chloride regulates inflammatory cytokine production in vitro. Relative messenger ribonucleic acid (mRNA) levels (fold changes) of interleukin-10 (IL-10), IL-6, IL-1β, and tumor necrosis factor-alpha (TNF-α) were examined with real-time polymerase chain reaction (PCR) in lithium chloride– (LiCl-) versus sodium chloride (NaCl)–treated murine macrophage-like RAW264.7 cells (**A**–**D**) and mouse bone marrow–derived neutrophils (**E**–**H**) before and at 6 h after *Pseudomonas aeruginosa* (PA) stimulation. The protein levels of IL-10 and TNF-α were examined with enzyme-linked immunoabsorbent assay (ELISA) in LiCl- versus NaCl-treated murine macrophage-like RAW264.7 cells (I) and mouse bone marrow–derived neutrophils (**J**) at 6 h after PA stimulation. Data are the mean±standard error of the mean (SEM) and represent three individual experiments (n=5). *, p<0.05; **, p<0.01; ***, p<0.001.

To ascertain the in vitro function of LiCl in infiltrating inflammatory cells, selected inflammatory cytokines were determined with real-time PCR in LiCl- versus NaCl-treated RAW264.7 cells and neutrophils. IL-10 expression was dramatically increased (Figure 5A, p<0.001), while the mRNA levels of IL-6 (Figure 5B, p<0.05) and TNF-α (Figure 5D, p<0.05) were decreased in the LiCl- versus NaCl-treated RAW264.7 cells after the PA challenge. No change was detected in IL-1β expression between the two groups (Figure 5C). Moreover, LiCl enhanced the expression of IL-10 (Figure 5E, p<0.01) and suppressed the expression of IL-6 (Figure 5F, p<0.05), IL-1β (Figure 5G, p<0.01), and TNF-α (Figure 5H, p<0.01) in PA-challenged neutrophils. Moreover, ELISA data showed that LiCl significantly upregulated IL-10 protein levels in RAW264.7 cells (Figure 5I, p<0.01) and neutrophils (Figure 5J, p<0.01), but downregulated TNF-α protein expression in RAW264.7 cells (Figure 5I, p<0.01) and neutrophils (Figure 5J, p<0.001).

### Lithium chloride promoted inflammatory cell apoptosis in vitro

**Figure 6:**
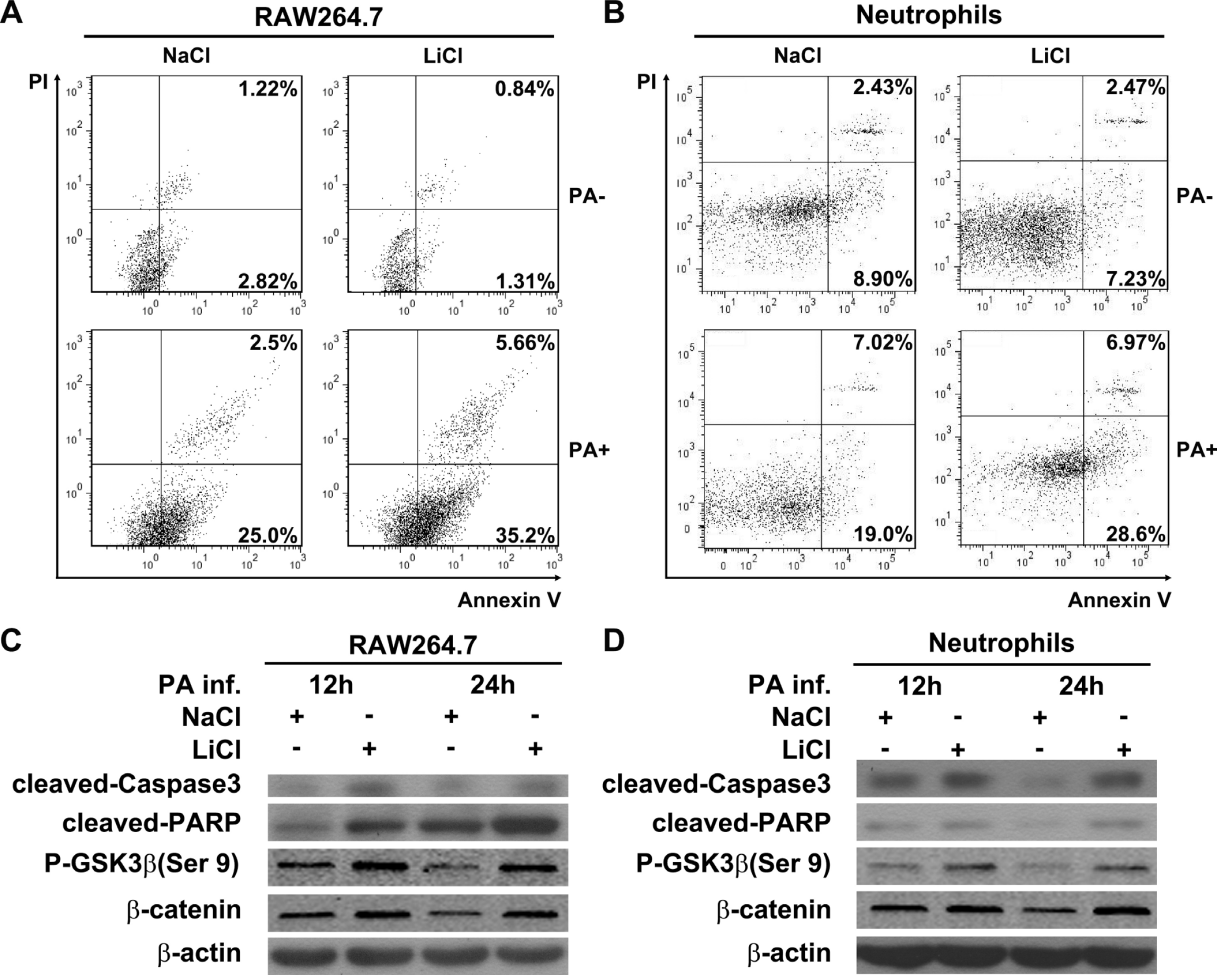
Lithium chloride promotes cell apoptosis in vitro. **A** and **B**: Apoptosis in lithium chloride– (LiCl-) versus sodium chloride– (NaCl-) treated murine macrophage-like RAW264.7 cells (**A**) and mouse bone marrow–derived neutrophils (**B**) before *Pseudomonas aeruginosa* (PA) infection (PA-) and after PA infection (PA+) was analyzed with flow cytometry with annexin V and propidium iodide (PI) staining. Western blot was used to detect the protein levels of cleaved caspase-3, cleaved poly(ADP-ribose) polymerase (PARP), P-GSK3β, and β-catenin in murine macrophage-like RAW264.7 cells (**C**) and bone marrow–derived neutrophils (**D**) at 12 and 24 h after PA infection. Data represent three individual experiments.

We further examined the role of LiCl in modulating apoptosis of inflammatory cells with annexin V/PI double staining associated with flow cytometry. Flow cytometry data showed that LiCl increased the number of annexin V–positive RAW264.7 cells (Figure 6A) and neutrophils (Figure 6B) after the PA challenge, while no change was detected in the LiCl- versus NaCl-treated cells without PA stimulation.

Additional evidence for the occurrence of apoptosis was obtained with western blot analysis for cleaved caspase-3 and cleaved PARP, two hallmarks of apoptosis. Western blot data showed that LiCl induced caspase-3 and PARP cleavage in RAW264.7 cells (Figure 6C) and neutrophils (Figure 6D) at 12 and 24 h after PA stimulation, when compared with the NaCl-treated cells, indicating that LiCl promoted the apoptosis of macrophages and neutrophils in response to PA challenge. Moreover, the efficacy of LiCl treatment in the RAW264.7 cells (Figure 6C) and neutrophils (Figure 6D) were confirmed with western blot, as indicated by the upregulation of P-GSK3β and β-catenin.

## Discussion

LiCl exerts an efficient role in treating various diseases, including Alzheimer disease [28], bipolar disorder [29], diabetes [30], and cancer [31]. However, the function of LiCl in microbial keratitis remains unclear. Our study demonstrated that LiCl promoted host resistance against PA keratitis by modulating pro- and anti-inflammatory cytokine production and inflammatory cell apoptosis, which shed light on the regulation of LiCl in ocular infection.

It has been reported that LiCl exhibits a potent anti-inflammatory effect in the pathogenesis of various diseases by modulating production of inflammatory cytokines. For example, Nahman et al. reported that LiCl relieves bipolar disorder by reducing the secretion of TNF-α, IL-1β, prostaglandin E2, and nitric oxide [19]. Beurel et al. demonstrated that LiCl reduces LPS-induced IL-6 expression in the septic shock murine model and in vitro cultured primary glia [39]. Wang et al. protected against endotoxemic acute renal failure mainly by downregulating proinflammatory TNF-α and Regulated on Activation, Normal T cell Expressed and Secreted [27]. Our study indicated that LiCl enhanced IL-10 production and reduced TNF-α expression in the infected corneas as well as murine macrophages and neutrophils. Moreover, LiCl also reduced expression of IL-6 in macrophages and IL-1β and IL-6 in neutrophils. Although the LiCl-modulated inflammatory cytokine profile in different cell types was not exactly the same, overall LiCl plays an anti-inflammatory role in the host immune response against PA infection. In addition to cytokine production, inflammatory infiltration is another hallmark of corneal inflammation. Our H&E staining data showed that at 5 days p.i., the LiCl-treated B6 corneas were much thinner and less swollen, with fewer infiltrated inflammatory cells in the stroma and anterior chamber, in contrast to the NaCl-treated corneas, further confirming the anti-inflammatory activity of LiCl.

Apoptosis is another strategy used by the host to control the excessive inflammatory response. Studies have demonstrated that LiCl promotes apoptosis of macrophages [22,40], neuron cells [23], and chick retina cells [25], but exhibits antiapoptotic activity in human acute T lymphoblastic leukemia cells [24], LPS-simulated renal cells [27], as well as transmissible gastroenteritis virus–infected swine testis cells and porcine kidney cells [26]. These data indicate that whether LiCl executes an antiapoptotic or a proapoptotic effect is largely associated with the cell type and stimulating factor. Our in vivo data demonstrated that in PA-infected B6 corneas, treatment with LiCl prompted the apoptosis of infiltrating neutrophils and macrophages, which together account for approximately 90% of the whole corneal cells. Our in vitro data further confirmed that LiCl exerts proapoptotic activity in murine macrophages and neutrophils after PA challenge, while no difference was detected in the LiCl- versus NaCl-treated cells before the challenge, which shed light on the regulation of LiCl in PA-induced inflammatory cell apoptosis.

According to previous studies, several mechanisms may be involved in the LiCl-induced anti-inflammatory responses and cell apoptosis. Studies have demonstrated that LiCl executes its immunomodulatory function by regulating the activity of enzymes involved in the second messenger system or signaling molecules such as GSK3β and β-catenin. For example, studies have demonstrated that LiCl induced the apoptosis of macrophages by suppressing inositol monophosphatase (IMPase) activity [22] and nuclear factor-kappaB activation [40], while the mechanism of neutrophil apoptosis induced by LiCl remains unclear. Substantive evidence indicated that in most cases, LiCl promotes anti-inflammatory cytokine production [23] and induces cell apoptosis by suppressing GSK3β activity [23]. However, in PA keratitis, specific GSK3β inhibitor SB216763 and LiCl displayed different effects in inflammatory cytokine expression, which indicated the participation of other signaling pathways in the LiCl-induced anti-inflammatory responses.

The role of inflammatory cell apoptosis in PA keratitis remains controversial. Dr. Hazlett’s group demonstrated that earlier apoptosis of inflammatory cells is beneficial for host immune defense against PA keratitis, and that interference of apoptosis aggravates tissue damage and enhances bacterial burden in PA-infected mouse corneas [9,10]. However, Dr. Pearlman’s group reported that PA promotes apoptosis of infiltrating neutrophils in the corneal stroma by virtue of the bacterial type III secretion system, and subverts the antibacterial activity of neutrophils [11]. Our study demonstrated that LiCl increased the apoptosis of infiltrating inflammatory cells and promoted host resistance against PA keratitis, which is consistent with Hazlett’s viewpoints. We also examined the microbicidal activity of LiCl by using the same murine model of PA keratitis, and found that LiCl promoted bacterial elimination, suggesting that the protective role of LiCl in PA keratitis depends on anti-inflammatory function and microbicidal activity.

In summary, our in vivo and in vitro studies provide substantive evidence that LiCl promotes host resistance to PA keratitis by suppressing inflammatory cytokine production, enhancing inflammatory cell apoptosis, and promoting bacterial clearance. Collectively, the data may hold promise for alternative clinical treatment of PA keratitis and other infectious diseases.
